# Reviving the Mitochondria: A Hopeful Horizon in Refractory Heart Failure

**DOI:** 10.7759/cureus.91710

**Published:** 2025-09-06

**Authors:** Zeeshan Ahmed, FNU Abdul Rehman, Syed Faqeer Hussain Bokhari

**Affiliations:** 1 Medicine and Surgery, King Edward Medical University, Lahore, PAK

**Keywords:** elamipretide, heart failure, mitochondira, mitoq, perhexiline, pgc-1α, trimetazidine, urolithin

## Abstract

Refractory heart failure (HF) remains a pressing clinical challenge despite substantial progress in pharmacologic and device-based interventions. While current therapies target neurohormonal, hemodynamic, and metabolic derangements, a crucial element of cellular dysfunction often remains overlooked: mitochondrial health. Mitochondrial dysfunction plays a central role in the pathogenesis and progression of HF, contributing to bioenergetic failure, oxidative stress, and cardiomyocyte death. Recent advances in mitochondrial-targeted therapies have opened new possibilities for treating HF at its energetic roots. This editorial explores the pathophysiological role of mitochondria in HF, reviews emerging therapeutic strategies aimed at restoring mitochondrial function, and outlines key challenges that must be addressed to translate these innovations into clinical practice.

## Editorial

Introduction

Heart failure (HF) represents one of the leading causes of hospitalization and mortality. Although therapies such as angiotensin-converting enzyme (ACE) inhibitors, beta-blockers, mineralocorticoid receptor antagonists, angiotensin receptor-neprilysin inhibitors (ARNIs), and sodium-glucose co-transporter 2 (SGLT2) inhibitors have substantially improved outcomes in many patients, a subset continues to deteriorate despite maximal therapy [1]. These individuals with refractory HF often experience recurrent hospitalizations, reduced exercise capacity, and declining quality of life, pointing to an urgent need for novel therapeutic targets [2].

Current pharmacologic approaches focus largely on modifying neurohormonal activation, reducing afterload, and mitigating congestion [3]. While these strategies alleviate symptoms and slow disease progression, they often fail to address the fundamental cellular-level impairments that drive ongoing myocardial dysfunction. Among these, mitochondrial abnormalities have gained increasing attention as both a marker and mediator of disease severity.

Central pathophysiologic axis

The heart is one of the most energy-demanding organs in the body, relying heavily on mitochondria to generate sufficient adenosine triphosphate (ATP) through oxidative phosphorylation. In a healthy adult heart, mitochondria contribute over 90% of the required ATP to sustain contractile function. In HF, however, mitochondrial function is significantly impaired, leading to a state of energy starvation and metabolic inflexibility [4].

Multiple mechanisms contribute to mitochondrial dysfunction in HF. One of the primary disturbances is impaired oxidative phosphorylation, which leads to a significant reduction in ATP production and compromises the energy supply of the heart. Additionally, failing mitochondria generate excessive reactive oxygen species (ROS), which cause oxidative damage to proteins, lipids, and mitochondrial DNA, further exacerbating cellular injury. Mitochondrial dynamics are also disrupted, with an imbalance between fission and fusion processes impairing the structural and functional integrity of the mitochondrial network. Moreover, defective mitophagy, the process responsible for the clearance of damaged mitochondria, leads to the accumulation of dysfunctional organelles within cardiomyocytes. Compounding these issues is mitochondrial calcium overload, which can prompt the opening of the mitochondrial permeability transition pore (mPTP), a critical event that initiates apoptosis and necrosis, contributing to progressive myocardial cell loss.

Importantly, these mitochondrial abnormalities are not merely byproducts of HF but active contributors to disease progression. Bioenergetic deficiency impairs myocardial contractility, while oxidative stress drives adverse remodeling, together creating a vicious cycle in which dysfunctional mitochondria continually exacerbate myocardial injury.

Clinical implications

Multiple lines of evidence support the role of mitochondrial dysfunction as a predictor of poor outcomes in HF [5]. Studies have demonstrated that patients with advanced HF exhibit lower mitochondrial respiratory capacity, decreased mitochondrial DNA content, and higher oxidative stress markers compared to healthy controls [5-7]. These abnormalities are often associated with reduced peak oxygen consumption (VO2 max), worse New York Heart Association (NYHA) functional class, and increased mortality risk. Despite these insights, no currently approved HF therapy directly targets mitochondrial pathology. Some agents, such as SGLT2 inhibitors, may exert indirect benefits on mitochondrial metabolism through effects on substrate utilization and redox balance, but these are not specific mitochondrial interventions. As a result, a significant portion of cellular-level dysfunction remains uncorrected, particularly in patients who remain symptomatic despite guideline-directed medical therapy (GDMT). Refractory HF may thus represent not only a failure of current systemic therapies but also a missed opportunity to address mitochondrial health as a root cause of ongoing dysfunction.

Emerging mitochondria-targeted therapeutics

With growing recognition of mitochondria as central regulators of myocardial health, the search for targeted therapeutics has gained considerable momentum. Multiple strategies are being investigated to restore mitochondrial integrity and function, ranging from membrane stabilization and ROS neutralization to metabolic reprogramming and enhancement of mitochondrial turnover. While most of these interventions are in early-stage development, several have already entered clinical trials, offering cautious optimism for their future use in patients with HF.

Elamipretide

Elamipretide is one of the most clinically advanced mitochondrial-targeted therapies currently in clinical development. It is a small, cell-permeable tetrapeptide (D-Arg-dimethylTyr-Lys-Phe-NH2) that selectively associates with cardiolipin, a phospholipid located on the inner mitochondrial membrane essential for maintaining cristae structure and electron transport chain stability. By binding to cardiolipin, elamipretide appears to improve mitochondrial respiration, reduce the generation of reactive oxygen species (ROS), and enhance ATP production. The Evaluation of Mitochondrial Effects on Reperfusion Against Cardiovascular Events-ST Elevation Myocardial Infarction (EMBRACE-STEMI) trial evaluated elamipretide in patients with anterior ST-elevation myocardial infarction undergoing primary percutaneous coronary intervention [6]. Although the primary endpoint (infarct size reduction) was not met, improvements in mitochondrial function and reductions in biomarkers of cardiac injury were observed. In the PROGRESS-HF trial, elamipretide was tested in chronic HF with reduced ejection fraction (HFrEF) [8]. The trial revealed trends toward improved exercise capacity and patient-reported outcomes, though statistical significance in change in left ventricular ejection fraction or stroke volume was not consistently achieved. The safety profile has been acceptable, encouraging further study in larger, event-driven trials.

Precision Redox Therapy

Oxidative stress has long been implicated in the pathogenesis of HF. However, systemic antioxidant therapies such as vitamin E and beta-carotene have failed to demonstrate clinical benefit, likely due to insufficient localization and disruption of physiological redox signaling. Mitochondria-targeted antioxidants address this limitation by selectively concentrating within the mitochondrial matrix, where most ROS are generated. One such compound, MitoQ, is a derivative of coenzyme Q10 linked to a lipophilic triphenylphosphonium (TPP+) cation, which facilitates mitochondrial uptake. Preclinical studies have shown that MitoQ reduces mitochondrial ROS, preserves mitochondrial DNA integrity, and improves endothelial function. In animal models of pressure overload-induced cardiac hypertrophy, MitoQ attenuated cardiac remodeling and fibrosis [9]. While human data in HF patients are limited, ongoing trials in other cardiometabolic conditions such as hypertension and chronic kidney disease may lay the groundwork for future application in HF. Other candidates in this class include SkQ1 and MitoTEMPOL, which are under investigation for similar roles.

Metabolic Modulators

In the failing heart, there is a shift from fatty acid oxidation (FAO) to less efficient glucose oxidation. However, this switch is often incomplete, leading to impaired metabolic flexibility and energy shortfall. Pharmacologic agents that optimize substrate utilization and reduce oxygen demand per ATP molecule can improve myocardial efficiency [10]. Two such drugs, trimetazidine and perhexiline, inhibit FAO enzymes, encouraging glucose oxidation instead.

Trimetazidine, an inhibitor of 3-ketoacyl-CoA thiolase, has demonstrated improvements in angina, left ventricular ejection fraction, and functional class in small HF trials. A meta-analysis of randomized controlled trials suggested a modest improvement in NYHA class and left ventricular ejection fraction (LVEF), although larger trials are needed for definitive conclusions [11]. Perhexiline, which inhibits carnitine palmitoyltransferase-1 (CPT1), has shown similar benefits but carries a narrow therapeutic window and risk of hepatotoxicity and peripheral neuropathy, limiting its use in clinical practice.

Mitophagy and Mitochondrial Biogenesis

Mitochondrial quality control is essential for maintaining a functional mitochondrial network. This includes mitophagy, the selective autophagic clearance of damaged mitochondria, and biogenesis, the creation of new, functional organelles. Dysregulation of these processes leads to the accumulation of dysfunctional mitochondria, contributing to progressive myocardial decline. Urolithin A, a natural metabolite produced by gut microbiota from ellagitannins (found in pomegranates), has been shown to induce mitophagy and improve mitochondrial health in aging skeletal muscle. A phase 1 trial demonstrated its safety and ability to enhance markers of mitochondrial function in humans [12]. Although its effects in cardiac tissue remain under investigation, its mechanism suggests potential utility in HF patients with impaired mitochondrial turnover.

PGC-1α

PGC-1α (peroxisome proliferator-activated receptor gamma coactivator 1-alpha) is a master regulator of mitochondrial biogenesis. Pharmacologic and genetic strategies aimed at upregulating PGC-1α expression have shown promise in preclinical models of HF [7]. Agents like AICAR (5-aminoimidazole-4-carboxamide ribonucleotide) and endurance-mimetic drugs are being explored to stimulate mitochondrial renewal and improve bioenergetic capacity. While enhancing mitophagy and biogenesis represents a sophisticated approach to mitochondrial therapy, most interventions remain preclinical. However, the mechanistic rationale is strong, and future translational studies may open this field further.

Novel Experimental Approaches

In addition to pharmacologic strategies, emerging concepts such as mitochondrial transplantation, gene therapy targeting mitochondrial DNA, and peptide-based mitochondrial delivery systems are under investigation. For instance, autologous mitochondrial transplantation into ischemic myocardium has shown feasibility in early preclinical models, with modest improvements in myocardial energetics [13]. Gene editing technologies such as clustered regularly interspaced short palindromic repeats (CRISPR)/Cas9 and mitochondrial-targeted transcription activator-like effector nucleases (mitoTALENs) are also being developed to address inherited mitochondrial DNA mutations. However, their role in acquired HF syndromes remains highly experimental. Moreover, nanoparticle-based delivery systems capable of targeting therapeutic agents specifically to cardiac mitochondria offer an exciting avenue to enhance drug efficacy and reduce systemic side effects. These approaches remain in their infancy but reflect the growing ambition to precisely intervene at the level of the mitochondrion (Table 1).

**Table 1:** Mitochondrial-Targeted Therapeutics in Heart Failure: Comprehensive Summary Table HF: Heart failure; LVEF: left ventricular ejection fraction; ATP: adenosine triphosphate; NYHA: New York Heart Association; CRISPR: clustered regularly interspaced short palindromic repeats; ROS: reactive oxygen species; EMBRACE-STEMI: The Evaluation of Mitochondrial Effects on Reperfusion Against Cardiovascular Events-ST Elevation Myocardial Infarction

Pharmacological Class	Drug/Agent	Mechanism of Action	Key Clinical Trial Results	Safety/Tolerability
Cardiolipin-targeting peptides	Elamipretide (D-Arg-dimethylTyr-Lys-Phe-NH2)	Small, cell-permeable tetrapeptide that selectively associates with cardiolipin on inner mitochondrial membrane; improves mitochondrial respiration, reduces ROS generation, enhances ATP production	EMBRACE-STEMI: Primary endpoint (infarct size reduction) not met, but improvements in mitochondrial function and reductions in biomarkers of cardiac injury observed [14].	Acceptable safety profile, encouraging further study in larger, event-driven trials
Mitochondria-targeted antioxidants	MitoQ (Mitoquinone)	Derivative of coenzyme Q10 linked to lipophilic triphenylphosphonium cation; facilitates mitochondrial uptake, reduces mitochondrial ROS, preserves mitochondrial DNA integrity, improves endothelial function	Preclinical studies showed reduced mitochondrial ROS, preserved mitochondrial DNA integrity, improved endothelial function. In animal models of pressure overload-induced cardiac hypertrophy, attenuated cardiac remodeling and fibrosis. Human data in HF patients limited; ongoing trials in hypertension and chronic kidney disease	Under investigation; preclinical safety data encouraging
	SkQ1	Mitochondria-targeted antioxidant	Similar roles to MitoQ under investigation	Under investigation
	MitoTEMPOL	Mitochondria-targeted antioxidant	Similar roles to MitoQ under investigation	Under investigation
Metabolic modulators	Trimetazidine	Inhibitor of 3-ketoacyl-CoA thiolase; inhibits fatty acid oxidation enzymes, encouraging glucose oxidation instead	Demonstrated improvements in angina, left ventricular ejection fraction, and functional class in small HF trials. Meta-analysis of randomized controlled trials suggested modest improvement in NYHA class and LVEF	Generally well-tolerated; larger trials needed for definitive conclusions
	Perhexiline	Inhibits carnitine palmitoyltransferase-1 (CPT1); promotes glucose over fatty acid oxidation	Similar benefits to trimetazidine demonstrated	Narrow therapeutic window; risk of hepatotoxicity and peripheral neuropathy, limiting clinical practice use
Mitophagy enhancers	Urolithin A	Natural metabolite produced by gut microbiota from ellagitannins (found in pomegranates); induces mitophagy and improves mitochondrial health	Phase 1 trial demonstrated safety and ability to enhance markers of mitochondrial function in humans; effects in cardiac tissue remain under investigation [12]	Safe in Phase I studies; mechanism suggests potential utility in HF patients with impaired mitochondrial turnover
Mitochondrial biogenesis stimulators	AICAR (5-Aminoimidazole-4-carboxamide ribonucleotide)	Stimulates mitochondrial renewal through endurance-mimetic pathway	Being explored to stimulate mitochondrial renewal and improve bioenergetic capacity	Under investigation
	PGC-1α targeting agents	Target master regulator of mitochondrial biogenesis (peroxisome proliferator-activated receptor gamma coactivator 1-alpha)	Pharmacologic and genetic strategies aimed at upregulating PGC-1α expression showed promise in preclinical models of HF	Under investigation; mechanistic rationale strong
Experimental approaches	Autologous Mitochondrial Transplantation	Direct transplantation of healthy mitochondria into ischemic myocardium	Showed feasibility in early preclinical models with modest improvements in myocardial energetics	Highly experimental; feasibility demonstrated but safety profile under investigation
	Gene Therapy (CRISPR/Cas9, mitoTALENs)	Gene editing technologies targeting mitochondrial DNA mutations	Being developed to address inherited mitochondrial DNA mutations; role in acquired HF syndromes remains highly experimental	Highly experimental
	Nanoparticle-based Delivery Systems	Targeted therapeutic agent delivery specifically to cardiac mitochondria	Offer avenue to enhance drug efficacy and reduce systemic side effects	Remain in infancy but reflect growing ambition for precise mitochondrial intervention

Challenges and future directions

While the therapeutic rationale for targeting mitochondria in HF is strong, several challenges must be overcome before these strategies can be broadly implemented. First, the HF population is highly heterogeneous, and mitochondrial dysfunction may vary significantly between individuals. Reliable biomarkers to stratify patients based on mitochondrial health are still lacking. Second, most current assessments of mitochondrial function require invasive tissue samples or specialized imaging techniques that are not routinely available in clinical practice. The development of non-invasive biomarkers, such as circulating mitochondrial DNA or metabolites of mitochondrial activity, could facilitate early identification of patients who would benefit from mitochondrial therapies. Third, long-term safety and tolerability of mitochondrial-targeted agents remain to be established. While many of these compounds show benefit in animal models, translating these effects into durable clinical outcomes requires well-powered randomized controlled trials with appropriate endpoints. Finally, an integrative approach that combines mitochondrial-directed therapies with existing GDMT may yield the most substantial benefits. Rather than replacing current treatments, mitochondrial therapies may serve as adjunctive strategies that address energy failure and cellular health in patients who do not fully respond to standard regimens.
